# Prevalence of influenza A virus in live-captured North Atlantic gray seals: a possible wild reservoir

**DOI:** 10.1038/emi.2016.77

**Published:** 2016-08-03

**Authors:** Wendy Blay Puryear, Mandy Keogh, Nichola Hill, Jerry Moxley, Elizabeth Josephson, Kimberly Ryan Davis, Chistopher Bandoro, Damian Lidgard, Andrea Bogomolni, Milton Levin, Shelley Lang, Michael Hammill, Don Bowen, David W Johnston, Tracy Romano, Gordon Waring, Jonathan Runstadler

**Affiliations:** 1 Massachusetts Institute of Technology, Cambridge, MA 02139, USA; 2 Mystic Aquarium, Mystic, CT 06355, USA; 3 Duke University, Beaufort, NC 28516, USA; 4 National Oceanic and Atmospheric Administration, Northeast Fisheries Science Center, Woods Hole, MA 02543, USA; 5 Dalhousie University, Halifax, Nova Scotia, Canada B3H 1C2; 6 Woods Hole Oceanographic Institution, Woods Hole, MA 02543, USA; 7 University of Connecticut, Storrs, CT 06268, USA; 8 Department of Fisheries and Oceans, Dartmouth, Nova Scotia, Canada B2Y 4A2

**Keywords:** gray seal, *Halichoerus grypus*, influenza, pinniped, reservoir, telemetry

## Abstract

Influenza A virus (IAV) has been associated with multiple unusual mortality events (UMEs) in North Atlantic pinnipeds, frequently attributed to spillover of virus from wild-bird reservoirs. To determine if endemic infection persists outside of UMEs, we undertook a multiyear investigation of IAV in healthy, live-captured Northwest Atlantic gray seals (*Halichoerus grypus*). From 2013 to 2015, we sampled 345 pups and 57 adults from Cape Cod, MA, USA and Nova Scotia, Canada consistently detecting IAV infection across all groups. There was an overall viral prevalence of 9.0% (95% confidence interval (CI): 6.4%–12.5%) in weaned pups and 5.3% (CI: 1.2%–14.6%) in adults, with seroprevalences of 19.3% (CI: 15.0%–24.5%) and 50% (CI: 33.7%–66.4%), respectively. Positive sera showed a broad reactivity to diverse influenza subtypes. IAV status did not correlate with measures of animal health nor impact animal movement or foraging. This study demonstrated that Northwest Atlantic gray seals are both permissive to and tolerant of diverse IAV, possibly representing an endemically infected wild reservoir population.

## INTRODUCTION

Since 1972, there have been several reports of influenza A virus (IAV) in seals around the globe, including multiple locations in the Americas and Eurasia, representing several seal species.^1, 2, 3, 4, 5, 6^ These observations have primarily been in response to unusual mortality events (UMEs) and have generally been regarded as dead-end spillover events. Harbor seals (*Phoca vitulina*) appear particularly susceptible to mass mortalities associated with diverse influenza infections, as evidenced by periodic outbreaks along the northeast coast of the United States in 1979 (H7N7), 1982 (H4N5 and H4N6), 1991 (H3N3) and 2011 (H3N8).^3, 7, 8, 9, 10^ Most recently (2014), H10N7 was associated with a mass mortality event in harbor seals of the North Sea.^11, 12, 13^

Although influenza-associated seal UMEs demonstrate that seals can serve as a spillover host, there are suggestions that the virus could be endemic in seal species.^14^ An *in vivo* study using experimental infection with a seal-derived H7N7 found that while harbor seals developed disease, gray seals and harp seals (*Pagophilus groenlandicus*) were resistant to disease,^7^ reminiscent of the low pathogenicity seen in avian natural reservoirs.^15^ Analyses of archived serology samples from many of the seals with an Arctic distribution (harp seals, ringed seals (*Phoca hispida*) and hooded seals (*Cystophora cristata*)) were seropositive without apparent signs of morbidity, again suggesting the possibility of endemic IAV infection.^3, 7, 8, 9^ Most recently, gray seals in the North Sea were found to have antibodies against H10N7 despite a lack of morbidity, and predating the H10N7-associated mortality event that followed soon after in harbor seals in the same region.^16^

Reservoir hosts are often defined as being a long-term host for a pathogen in which infection is well tolerated and maintained at a subclinical level.^17, 18, 19^ As a reservoir host does not typically show signs of disease, it provides a mechanism for the pathogen to be maintained and evolve, and from which spillover into new populations can occur. In contrast, a spillover host is typically exemplified by strong and acute immune activation, disease progression and morbidity or mortality.^17^ Ongoing endemic infection is a key indicator of a reservoir host, particularly when coupled with asymptomatic infection and minimal long-term fitness costs. Mammalian reservoir hosts are of particular importance in that they can provide a mixing vessel and/or source of mammalian adaptation for IAV. When avian-specific variants acquire the ability to infect mammalian hosts, either through incremental mutations, or genomic reassortments brought about by coinfection with multiple viruses, there is a risk for human pandemic infection.^20, 21, 22^ As epizootic instances become more frequent, understanding the dynamics involved in interspecies transmission becomes increasingly important.

The changing ecology of gray seals possesses several factors that make them an intriguing species to investigate as a potential influenza reservoir host. First, gray seal numbers declined in both eastern and western Atlantic waters, largely due to human interactions. Recently, their populations have rebounded, due in large part to the Marine Mammal Protection Act (1972), leading to re-colonization and increased population density across the North Atlantic, with steady increases noted in New England waters, Sable Island, Nova Scotia (NS), the United Kingdom and the Wadden Sea.^23, 24, 25, 26, 27, 28^ Host populations that occur at high density are associated with an increase in infection risk, a pattern consistent across wildlife species.^29^ Second, gray seals appear to be fairly resistant to infectious disease caused by viral agents, in contrast to the harbor seals that share much of their range.^3, 11, 30, 31^ The absence of discernable morbidity brought about by infection is expected to enhance the probability of the virus being maintained within the population. Third, gray seals are highly social and interact with one another regularly and intimately, hauling out on top of one another, engaging in open mouth aggression, competing for mates, and hissing and spitting at one another, all interactions expected to favor pathogen transmission.^27, 32, 33^ Finally, there is mounting evidence to suggest that gray seals attack other gray seals,^34^ harbor seals,^35^ harbor porpoises^36, 37^ and seabirds,^38, 39^ interactions that provide opportunities for cross-species transmission. If gray seals serve as an underappreciated natural reservoir for IAV, increasing populations may result in amplification and dissemination of virus along the coast to other mammals, coastal birds and potentially seeding of novel human infections.

## MATERIALS AND METHODS

### Surveillance sites

The largest gray seal colonies in North America were targeted for longitudinal sampling and included breeding colonies in Canada and the United States (US) (Table 1). All US sampling was on Cape Cod, MA, with pups sampled at Muskeget island (41.334 N, 70.293685 W) and Monomoy Island (41.559 N, −69.993 W), and adults sampled at Chatham (41.704 N, −69.937 W). All work within US waters was performed under National Marine Fisheries Service (NMFS) permit numbers #10080-95 or 17670-01. Work on Monomoy island was performed under a National Wildlife Refuge (NWR) System Research and Monitoring Special Use Permit, while samples from Sable Island, Canada were received through NMFS #17298-01. The majority of weaned pups were sampled on Cape Cod during January in the breeding seasons of 2013, 2014 and 2015; a small number of pups were sampled on Sable Island during January of 2014. Adults were sampled at both Cape Cod and Sable Island during the summer of 2013, and a subset at each location were cellular or satellite tagged.

#### Muskeget and Monomoy Islands, United States

Weaned gray seal pups were sampled on Muskeget Island in 2013, 2014 and 2015, and on Monomoy Island, Cape Cod in 2015. There was no significant difference between the two sites in the number of animals found to be IAV positive by either serology or quantitative real-time polymerase chain reaction (qPCR; Fisher's exact two-tail *P*>0.1) during the 2015 season; the data from that year was therefore pooled for analysis. Muskeget is an uninhabited island located just west of Nantucket and Tuckernuck islands off Cape Cod, MA, USA. It comprises 292 acres of sand dunes, marsh, and two fresh water ponds, and is both the southernmost and largest US breeding colony of gray seals.^25^ Monomoy NWR is located on the southeast corner of Cape Cod near Chatham, MA and encompasses 7604 acres of sand dunes, marsh and freshwater ponds that provide critical habitat to numerous migratory and endangered birds. A gray seal breeding colony became established on the South Monomoy Island in the early 1990s and has continued to grow, currently representing the second largest gray seal breeding site in the United States after Muskeget Island. In addition to gray seals, harbor, harp and occasional hooded seals haul out on the island. Monomoy is also home to additional mammals, such as coyotes, deer, muskrats and voles, provides habitat to over 300 bird species and is an important nesting area for migratory shorebirds including federally protected piping plovers and roseate terns. Muskeget and Monomoy are ~18 nautical miles apart.

#### Nova scotia, canada

Sable Island, Nova Scotia, Canada, is the largest breeding colony for Northwest Atlantic gray seals.^25^ It is located 190 miles southeast of Halifax and is a narrow stretch of land, 26 miles long and <1 mile wide. It is ~460 nautical miles from Monomoy Island, USA. Sable Island is also a migratory bird sanctuary with over 200 bird species, serving as a breeding area for common, arctic and roseate terns, great black-backed gulls, herring gulls and the Ipswich sparrow. The only additional mammalian residents are feral horses and harbor seals.

### Animal handling and sample collection

Weaned gray seal pups were captured on land and were physically restrained during sampling and monitored for signs of distress. Adult gray seals at Cape Cod were captured by purse seine, carefully pulled ashore and sampled under sedation; adult seals on Sable island were captured on land using a hinged pole net and restrained for ~15 min without sedation. Morphometrics were taken on each animal, including weight, straight length, axial girth and flipper length. As a proxy for age, pups were coded for molt stage ranging from II (youngest) to V (oldest): stage II, weaned with complete lanugo; stage III, molted around face and flippers; stage IV, molting on body; stage V, completed molting.^40^ Cattle tags were placed through the webbing of one or both hind flippers for later identification. Sterile polyester tipped swabs were used to collect conjunctival, nasal and rectal swab samples from each animal. Conjunctival samples were obtained from both eyes by swabbing the medial sclera lateral to the lacrimal caruncle of the eye and in the inside rim of the lower eyelid where possible. Nasal samples were obtained by swabbing rapidly in the inside of each nostril and rotating the swab two to three times. Rectal samples were obtained by inserting the swab into the rectum with gentle pressure and rotating the swab two to three times. Swabs were placed into viral transport media (VTM) (M4RT from Remel Inc., Lenexa, KS, USA) and kept chilled for up to 8 h in the field before being stored below −80 °C. Approximately 10 mL of blood (<1 mL/kg) was collected using standard aseptic techniques from the extradural intravertebral vein using a 20G 1.5 in to 3.5 in needle. Blood samples were stored chilled for up to 8 h until further processing. Serum tubes were centrifuged to separate serum and stored below −80 °C. EDTA-treated blood was collected from a subset of pups from 2013 and analyzed for complete blood cell counts (CBCs) using a Veterinary Hematological System (Heska Corporation, Loveland, CO, USA). Hematocrit was determined using a standard clinical microhematocrit centrifuge (BD Triac Centrifuge, Franklin Lakes, NJ, USA). Total white blood counts (WBCs) were measured and used to calculate the mean corpuscular volume, mean corpuscular hemoglobin and mean corpuscular hemoglobin concentration.

### Telemetry

In 2013, nine adult gray seals were tagged in June at Chatham, MA, USA: seven using global positioning system (GPS) tags and two using satellite tags, one of which was provided through whalenet.org (Michael Williamson, Wheelock College/Whalenet to Northwest Atlantic Seal Research Consortium (NASRC)) and the other provided by the Northeast Fisheries Science Center (NEFSC).^41^ In each of 2013 and 2014, 12 adult gray seals were tagged on Sable Island, NS. Each seal was fitted with a Mk10-AF Fastloc GPS tag (Wildlife Computers, www.wildlifecomputers.com) programmed to transmit Advanced Research and Global Observation Satellite (ARGOS) and GPS data and to archive GPS data that were downloaded on recovery of the tag.^42^ The tags possessed a variety of sensors to collect high-resolution movement and dive behavior, and were attached to the dorsal neck/head region of the animal's fur using an epoxy-based adhesive.^41^ Each tag measured 10 cm × 7 cm × 4 cm and weighed 370 g (in air). GPS tags: GSM/GPRS devices (Global System for Mobile Communications/General Packet Radio System, Sea Mammal Research Unit (SMRU) Instrumentation, St Andrews, Scotland) archive the data during at-sea periods and transfer using available GSM mobile phone networks upon return to the beach and haul out. Satellite tags: transmit data to the ARGOS System in Largo, MD, USA whenever an animal surfaces.

### Detection of virus

Viral RNA was extracted from 50 μL of VTM sample using the Omega Mag-Bind Viral DNA/RNA Kit (Omega Bio-Tek, Norcross, GA, USA) and a KingFisher Magnetic Particle Processor (Thermo Scientific, Waltham, MA, USA). RNA was screened using qScript XLT One-Step RT-qPCR ToughMix (Quanta Biosciences, Gaithersburg, MD, USA) and analyzed for fluorescence on an ABI 7500 real-time PCR System (Applied Biosystems, Foster City, CA, USA). A conserved region of the matrix gene was targeted using two separate primer sets optimized for avian isolates^43^ and mammalian isolates.^44^ Samples were batch processed with each 96-well plate containing two positive and eight negative controls. Any sample with a cycle threshold (Ct) value <45 was considered positive, as recommended by the United States Department of Agriculture (USDA) for influenza screening in wild birds.^45^ An animal was considered positive for active viral shedding if any of the three swabs (nares, conjunctiva, rectal) were avian influenza (AI) matrix positive. The average Ct value of positive swab samples was 38.21 (range 32.45–42.0). Only one sample had a Ct <35, the cutoff frequently required for potential virus isolation.^46, 47, 48, 49^ Despite high Ct values, all AI matrix positive samples were passaged in embryonated chicken eggs (ECEs) (Charles River, CT, USA) and a subset were tested in Madin–Darby canine kidney (MDCK) cells. As expected with high Ct values, the majority of samples failed to grow; those that grew remained at a low concentration (Ct >35) and failed to generate sequence by both Sanger and next-generation sequencing.

### Serology and hemagglutination inhibition

Serum collected from seals were screened for influenza antibodies using an enzyme-linked immunosorbent assay (ELISA) kit (IDEXX AI MultiS-Screen, Westbrook, ME, USA) following the manufacturer's instructions. Each sample was run in duplicate using 10 μL per well, diluted 1:10 with sample buffer. Positive and negative controls were run on each plate. Absorbance values were measured with an Epoch Spectrophotometer (BioTek Instruments, Winooski, VT, USA) and the average for each sample was used to calculate the sample to negative ratio. Samples with a ratio ≤0.6 were considered positive for influenza antibodies.^50, 51^

Seropositive samples were tested for the ability to inhibit hemagglutination from a panel of influenza A isolates in a protocol adapted from.^52, 53, 54^ Serum was diluted 1:4 with receptor destroying enzyme (RDE), incubated 18–20 h at 37 °C, then 30 min at 56 °C. Positive control reference sera was obtained from the Influenza Research Database and Centers of Excellence for Influenza Research and Surveillance (CEIRS) proficiency panel sera. Treated sera was serially diluted two-fold in phosphate-buffered solution and incubated with four hemagglutination units (HAU) of virus for 60 min at room temperature (RT). To verify effective RDE treatment and the absence of nonspecific hemagglutination, each treated sera was also tested for hemagglutination in the absence of virus. A final concentration of 0.25% chicken erythrocytes was added for an additional 20–60 min at RT. The hemagglutination inhibition (HI) titer was recorded as the last dilution where complete inhibition occurred, as evidenced by the presence of buttoned erythrocytes. Serum was defined as negative for HI titers below 1:8, moderate for HI 1:16–1:64, and strong for HI titers >1:128. The virus panel included human-derived isolate: pH1N1 (A/Netherlands/2629/2009/H1N1pdm), harbor seal-derived isolate H3N8 (A/harbor seal/New Hampshire/179629/2011/H3N8) and avian-derived isolates H2N3 (A/duck/Interior Alaska/11PG00459/2011/H2N3), H3N8 (A/mallard/Interior Alaska/10BM11414R0/2010/H3N8), H4N6 (A/mallard/Interior Alaska/11BM01180/2011/H4N6), H6 (A/goose/Interior Alaska/11PG00149/2011), H7N3 (A/mallard/Interior Alaska/10BM08884/2010/H7N3), H8N4 (A/northern pintail/Interior Alaska/9BM11643/2009/H8N4), H9N2 (A/mallard/Interior Alaska/10BM02980/2010/H9N2), H10N6 (A/mallard/Interior Alaska/10BM16203/2010/H10N6), H11N9 (A/glaucous-winged gull/Southeastern Alaska/10JR01856/2010/H11N9), H12N5 (A/mallard/Interior Alaska/11BM01009/2011/H12N5), H13N6 (A/ring-billed gull/Massachusetts/12DC00060/2012/H13N6) and H16N2 (A/glaucous-winged gull/Southcentral Alaska/11JR00243/2011/H16N2). All viral stocks were grown in ECEs and avian allantoic fluid was titered to obtain HAU. The following serum positive samples were analyzed: 2013 pups (*n*=9), 2014 pups (*n*=11), 2015 pups (*n*=8), 2013 adults from Cape Cod (*n*=6) and 2013 adults from Sable island (*n*=6).

### Statistical analysis

For each prevalence estimate, standard error was calculated as 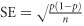, where *p*=prevalence and *n*=individuals sampled. Each sampling group represented <10% of the total population. To identify whether infection of seals was influenced by age (immature, mature), sex (male, female), sampling location (USA, Canada), year (2013, 2014, 2015) or season (winter, summer), partial least squares (PLS) regression was performed. PLS was selected as the model for testing due to the highly correlated nature of the predictor variables. Correlation matrices indicated that age and season, in particular, were highly correlated. Three infection metrics were tested with PLS that were defined by two or four outcomes: virus shedding (positive/negative), seroprevalence (seropositive/seronegative) and infection status (virus positive–seropositive, virus positive–seronegative, virus negative–seropositive and virus negative–seronegative). Infection metrics were treated as the nominal response variable. Age classes were pooled into immature (pups, yearlings and subadults) and mature (adults) to reflect breeding maturity of seals. PLS indicated which ecological variables were important in explaining variation observed in each of the three response variables. If an ecological variable had a small variable importance projection (VIP) of <0.8,^55^ it was considered a weak predictor of seal infection. The relative importance of each variable was assessed by ranking the total effect size of each parameter. Variable importance estimates assessed whether variability of the dependent/response factor was a function of variability associated with each parameter.^56^ This process relied on Monte Carlo resampling of observed values due to the assumption of non-uniformity of seal sampling across space and time. All statistics were performed using JMP Pro 12.1 for Macintosh.

## RESULTS

### Northwestern Atlantic gray seals show recurring evidence of both active and cleared influenza A Virus

#### Animals sampled

A total of 402 Northwest Atlantic gray seals were sampled from both Sable Island, NS, Canada and Cape Cod, MA, USA. The majority of animals (340/402) were sampled on Cape Cod and comprised predominantly pups (345/402) from the January breeding seasons of 2013 to 2015. Sampling effort based on season, year, age and location are shown in Table 1.

#### Viral shedding

**Figure 1:**
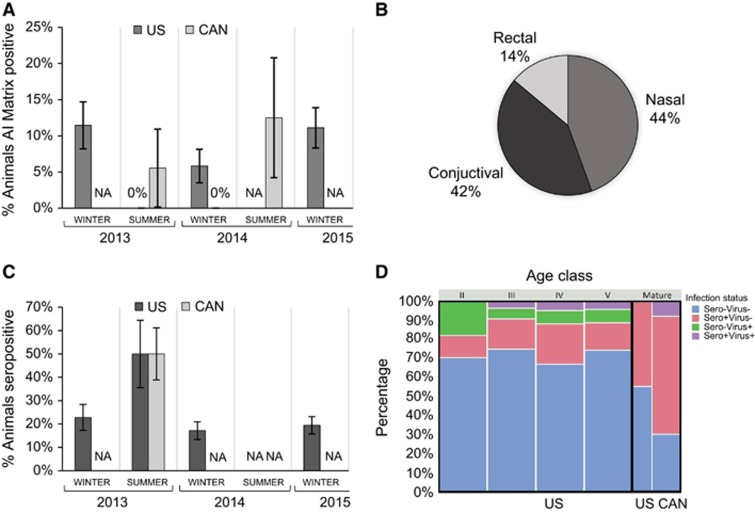
Influenza prevalence by time and location; USA sampling is graphed in dark gray, Canadian (CAN) sampling is graphed in light gray. Absence of virus is marked at 0% absence of sampling is marked as not applicable (NA). Each data set is derived from a range of 8–126 individuals, bars represent ±SE of the prevalence estimate (**A**, **C**). The percentage of animals where AI was detected in at least one of the three swab sites is shown in **A**; percentage of seropositive animals is shown in **C**. The proportion of positive samples derived from each physiologic site (rectal, conjunctival and nasal) is shown in **B**. Animals are grouped by infection status based on seroprevalence (sero) and detection of viral RNA (virus): negative by both serology and RTPCR (blue), positive by both serology and RTPCR (purple), seropositive and RTPCR negative (pink), seronegative and RTPCR positive (green). The proportion of animals in each infection status are shown based on age class (pup molt stages II–V, vs mature animals), relative sampling effort per group is represented by the width of the bars (**D**).

Overall, 9.0% (confidence interval (CI): 6.4%–12.5%) of the gray seal pups and 5.3% (CI: 1.2%–14.6%) of the adults were AI matrix positive. Prevalence varied across years, with pup prevalence levels in 2014 trending lower than both 2013 and 2015, though not statistically significant. The difference across sampling years suggests inter-annual variability (Figure 1A, dark gray bars), a trend supported by a PLS model wherein sampling year was a strong predictor of viral shedding (2014 VIP=1.73; 2015 VIP=2.04). Within a season, there was evidence that sample location also had a role in prevalence; during the summer of 2013 animals sampled in Canada were found to be AI matrix positive (Figure 1A, light gray bars) while those in the USA waters during 2013 were not; the opposite trend was observed during the winter of 2014, wherein animals from the USA had detectable virus and those from Canada did not (sampling location VIP=1.03). Of all IAV positive samples, the majority were detected from nasal (44.4%) and conjunctival (41.7%) swabs, with only a small fraction of total IAV derived from rectal swabs (13.9%) (Figure 1B).

#### Seroprevalence

The majority of animals were also screened for IAV-specific antibodies directed against a conserved epitope of the IAV nucleoprotein (NP). Combined data across the three years of sampled pups on Cape Cod showed 19.3% (CI: 15.0%–24.5%) were seropositive, a level consistent throughout the three winters sampled (Figure 1C). A smaller sampling of adults was analyzed in the summer of 2013 and included 12 animals from Cape Cod, USA and 20 animals from Sable Island, Canada; seroprevalence was 50% at each location (CI: 25.4%–74.6% Cape Cod and 29.9%–70.1% Sable Island). A PLS statistical model suggests that season (winter vs summer), age (immature vs mature) and sampling region (Cape vs Sable), all show robust effect sizes (VIP values of 1.37, 1.25 and 1.21, respectively) that warrant closer analyses with a larger sample size.

#### Infection status

A total of 92/402 animals (22.9%, CI: 19.0%–27.3%) showed evidence of IAV infection (current or past) from one or both screening methods. Grouping the animals by age class revealed distinct trends in group infection status associated with location (USA or Canada) and age (pup molt stages II–V vs mature breeding status; Figure 1D). Although nearly 75% of the pups from Cape Cod were negative for IAV, the remaining animals comprised those presumed to be in acute infection (virus+, sero−), peak infection (virus+, sero+) and either late infection or carrying maternally derived antibodies (virus−, sero+). Given that the pups in this study were recently weaned, the fraction of pups that were identified as virus−/sero+ could either represent animals where infection had already cleared, or animals where passive maternal antibodies from a seropositive dam have not yet fully decayed. To further decipher this dichotomy and to infer probable windows of viral acquisition, we used molt stage as a proxy for pup age and grouped the animals into stages II, III, IV and V^40^ (Figure 1D). Acute infection (sero−/virus+) was detected in all ages (Figure 1D: green), though represented the highest proportion for the most recently weaned pups (stage II). Peak infection represented a small percentage, but only occurred in the older pups (stages III–V). Animals that were sero+/virus− occurred in all stages, including the youngest most recently weaned pups (stage II).

In contrast, none of the mature animals that we tested from either Cape Cod or Sable Island, showed any evidence of acute infection (virus+, sero−), and while both mature populations showed evidence of past exposure (virus−, sero+), only those on Sable Island had any viral shedding (virus+, sero+). PLS analyses identified season, age and location as important variables (VIP of 1.39, 1.26 and 1.18, respectively).

### Influenza A infection in gray seals is asymptomatic and has minimal fitness costs

#### Health assessments

**Figure 2:**
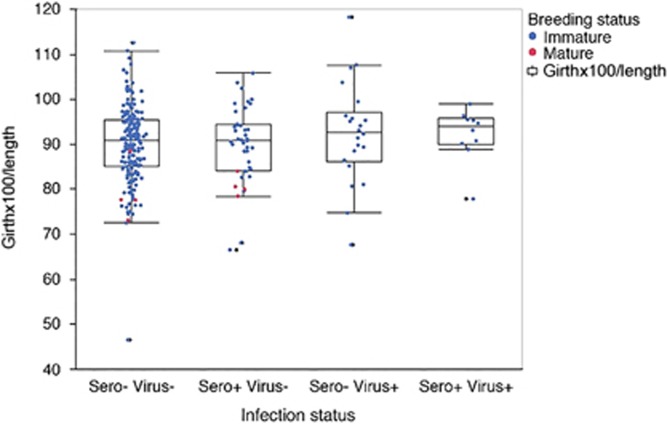
Animals are grouped by infection status based on seroprevalence (sero) and detection of viral RNA (virus). Graphed from left to right are sero/virus −/−, +/−, −/+, +/+. Each animal is shown as a single data point representing the Smirnov body condition index of girth × 100/length; immature animals (blue), mature animals (red). Whisker and box plot represent 5th and 95th quartiles.

In order to approximate whether or not IAV infection had an impact on the overall health of the animal, a Smirnov body condition index was applied (girth × 100/length). There were no significant differences in body condition associated with any of the four infection statuses (one-way analysis of variance, *F* ratio=0.7057, *P* =0.55, Figure 2).

In 2013, blood samples from a subset of animals were analyzed for CBCs. Of the 25 animals screened, five were seropositive by ELISA, and of those five, two also had detectable viral RNA. The seropositive animals were matched to seronegative animals according to gender and molt stage; the seropositive animals had evidence of slightly elevated WBC as compared with matched seronegative animals (10.9 vs 8.2, respectively). However, all samples fell within the normal healthy range for gray seal pups and the observed difference was not statistically significant (two-tailed *t-*test, *P*=0.08).^57^

#### Movement of animals tagged on cape cod

**Figure 3:**
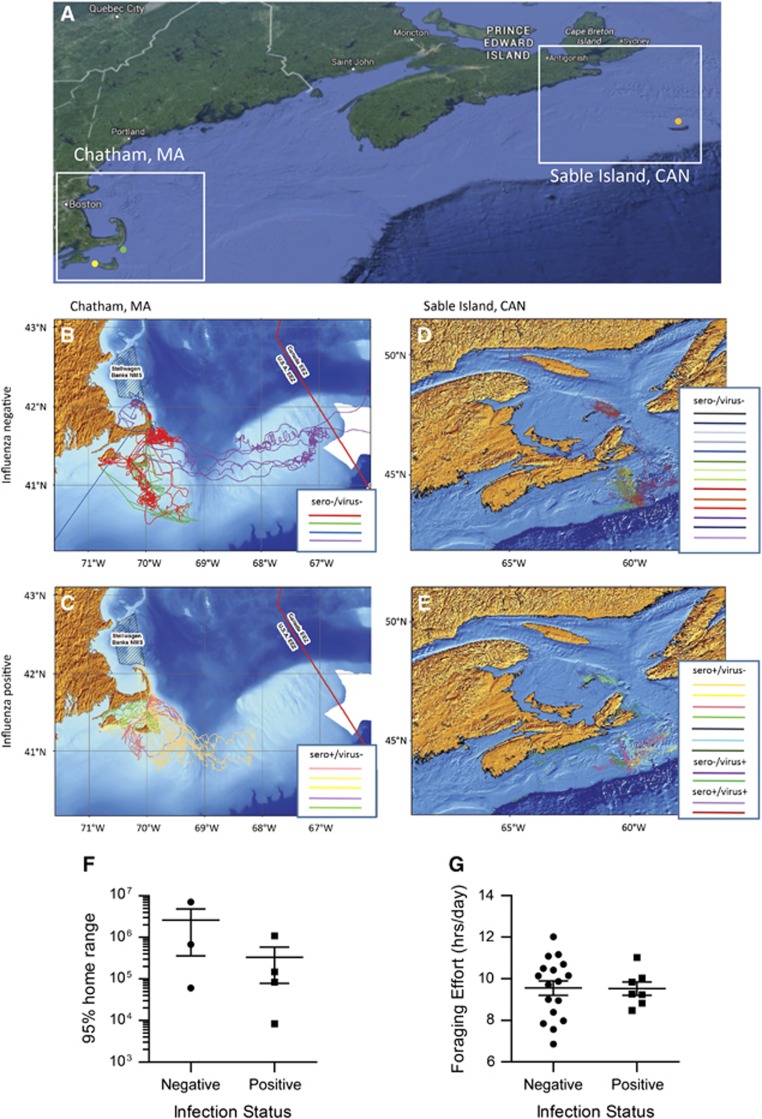
Movement of IAV positive and negative adult gray seals tagged in the summer of 2013 at either Sable Island, Canada (orange dot) or Chatham, MA (green dot). Additional animals were sampled without tagging on Muskeget, MA (yellow dot) (**A**). For each location, Chatham, MA (**B**, **C**) and Sable Island, Canada (**D**, **E**) the movement data for animals testing negative for influenza are shown on the top (**B**, **D**) and those testing positive are shown on the bottom (**C**, **E**). Tagged animals at each location were analyzed according to infection status: negative, or positive (sero+/virus−, sero−/virus+, sero+/virus+ denoted on the legend) (**F**, **G**). The 95% home range for animals tagged at Chatham, MA is shown as a scatter plot denoting the mean and standard error of the mean. (**F**). The foraging effort (FE) of positive and negative animals is shown as a scatter plot (**G**); the mean and SEM are shown. FE is calculated as hours at the lowest depth divided by sampling days.

Adult gray seals were tagged in Chatham, MA, USA near the Monomoy NWR headquarters (Figure 3A, green dot). This is the closest mainland point to Monomoy Island, where pup sampling occurred. Of the nine tagged animals, five were seropositive for IAV at the time they were tagged in June. Movement data from November 2013 through March 2014 of seronegative and seropositive animals is shown in Figures 3B and 3C, respectively. All animals made extensive movements around the Cape Cod region and dispersed amongst regional haul-outs, particularly in the months prior and immediately following breeding (December–February). One animal showed long-range movements from southern Cape Cod to the Gulf of Maine, Great South Channel, Georges Bank, and close to Sable Island. The seronegative animals trended toward a larger 95% home range, showing a median range of 671 701 km over the study period, while seropositive animals covered a median 116 044 km during the same period. However the small sample size revealed no statistical differences in home range based on serostatus (Figure 3F). Similarly, the seronegative animals trended toward further median summer travel distances than did the seropositive animals (2386 and 1411 km, respectively between June and November 2013), and further median winter travel distances (5268 and 3894 km, respectively between November 2013 and March 2014). There were no statistical differences in total travel distance based on serostatus (two-tailed *t*-test, *P*<0.05).

#### Movement of animals tagged on sable island

On Sable Island, 12 adult gray seals were tagged in 2013 and 12 additional animals were tagged in 2014. Seven individuals were positive for influenza, including four animals that were seropositive but virus negative, one seropositive animal that was also virus positive, and two animals actively shedding virus that had not yet seroconverted. Because of the limited number of virus positive animals, animals with any evidence of IAV infection past or present were grouped as positive for the purpose of mapping. Figures 3D and 3E show that the movements of the negative and positive animals are remarkably similar. In order to investigate if IAV infection had any discernable effect on diving and foraging behavior, the negative and positive animals were compared for foraging effort (Figure 3G). Foraging effort is defined as the number of hours spent at the bottom of a dive, divided by the number of sampling days.^58, 59^ No significant differences were observed.

### Seropositive gray seals respond to diverse subtypes of influenza A virus

**Figure 4:**
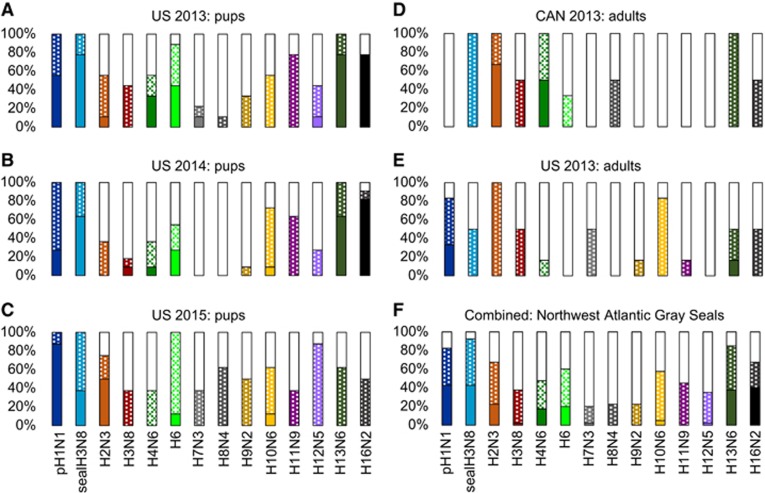
Sera from seropositive animals were tested for the ability to inhibit virus-driven hemagglutination of chicken erythrocytes. The virus panel represents human pH1N1, seal H3N8, and 12 avian viruses. For each subtype, the proportion of animals that failed to inhibit the virus are shown in white (≤1:8), moderate inhibition as a dotted pattern (1:16–1:64), and strong inhibition as a solid bar (≥1:128). Animals are grouped by sampling year (2013, 2014 or 2015) and location (Cape Cod, MA, USA or Sable Island, Canada) (**A**–**E**). Combined data for all animals tested is shown in **F**.

Overall, there was a surprisingly broad response in the 28 pups and 12 adults tested for the ability to inhibit hemagglutination against the panel of influenza A viruses (Figures 4A–4F). All viruses tested in this panel had at least one animal whose sera was able to inhibit virus at moderate levels (Figure 4F). Many of the panel viruses were inhibited by only a few sera (for example, H7N3 and H9N2) or at overall low to moderate levels (for example, H10N6). All pups from Cape Cod across all three years had sera that cross reacted with the 2011 harbor seal-derived H3N8, with many animals showing a strong response (Figures 4A–4C). Although many of the adult sera also cross reacted with seal H3N8, the response was more moderate than that seen in pups, and not all animals responded (Figures 4D and 4E). All pup sera tested from Cape Cod also cross reacted with pH1N1, predominantly giving a strong response. Adult sera from Cape Cod 2013 also responded strongly to pH1N1, while there was a complete absence of response to pH1N1 in sera from adults on Sable Island in 2013. In 2013 and 2014, the majority of pup sera from Cape Cod had strong cross reactivity to gull-associated subtypes H13 and H16. This cross reaction was also evident in adult sera and pup sera from 2015, but to a lesser degree.

## DISCUSSION

Three years of data spanning two geographic locations, breeding and non-breeding seasons, and immature and mature animals, demonstrate that Northwest Atlantic gray seals are at a minimum repeatedly infected by influenza A virus, and could also potentially act as a reservoir host. Approximately 5%–12% of animals were consistently found to have detectable viral RNA, with seroprevalence ranging from 19% in pups to 50% in adults. The degree of infection and exposure seen within this population is comparable to that seen in some of the wild birds that are widely accepted as reservoir hosts.^60^ Similarly, as with wild-bird reservoirs, the presence of IAV in gray seals does not appear to readily cause morbidity or mortality and IAV has never been reported in association with a dead stranded gray seal. However given that live virus was not recovered from the animals in this study, the extent to which virus is actively replicating remains unclear.

In a reservoir population, the young immunologically naive animals experience the highest percentage of acute infections, while mature animals benefit from an immunological memory response and demonstrate a high percentage of immunity. This trend was observed in the gray seal population, wherein acute infection was primarily detected in pups while the adult gray seals had a higher percentage of seropositive animals. Pups estimated to be only a few weeks to two months old were already showing acute infection, suggesting possible vertical transmission. However, horizontal transmission is also highly probable within the population, particularly in the older pups that are largely segregated from their mothers and other adults. A number of recently weaned pups presumed to be within a month of age had detectable antibodies, but no active viral shedding. As it is unlikely that the pups acquired virus and cleared infection within such a short time span, this likely reflects maternal antibody transfer. Such an interpretation is also consistent with our observation that 50% of adults were seropositive.

As predicted for a reservoir host, we found no evidence of pathogenicity within the IAV positive wild capture gray seals identified in this study. Body condition index is frequently used as an overall measure of animal health, with the Smirnov index providing a reasonable, albeit imperfect, metric for pinnipeds. We found no difference in the body condition of animals that were positive for IAV. We also found no difference in total white blood cells based on infection status, and all animals tested were found to be within the normal range for this species. However, the sample size for these analyses were small and the body condition metric can be difficult to interpret, particularly with pups that are fasting.

An additional metric of a pathogen-associated fitness burden is animal behavior and movement. Seals forage throughout the Gulf of Maine and visit multiple haul-out sites within their range. Both the Cape Cod and Sable Island populations reported in this study are of the same genetic stock^61^ and have been observed to travel between the two locations, with juveniles believed to exhibit a greater dispersal than adults. In May of 2015, a juvenile seal tagged in the Gulf of St Lawrence, Canada was reported ~800 miles away on Nantucket Island, Cape Cod. In addition, the Northwest Atlantic Seal Research Consortium recently constructed a database to report sightings of tagged animals (http://main.who.edu); multiple gray seals branded on Sable Island have been reported through this mechanism in Maine and around Cape Cod. To determine if IAV infection impacted regional or long-term movements, we opportunistically utilized GPS and satellite telemetry from a subset of animals. These animals were monitored prior to, during and after the breeding season. IAV infection status had no impact on how far the animals traveled nor the regions that they visited. Both seronegative and seropositive animals were found to make extensive regional movements and visit multiple haulout sites. Each of the tagged females within this study spent a prolonged period of time on Muskeget Island during the expected pupping period, suggesting these females were breeding/pupping, regardless of IAV status. There was one provisioned animal with restricted movements, and one animal that fell prey to a great white shark. However, both of these animals tested seronegative, so IAV infection did not influence the outcome.

We were unable to obtain sufficient viral RNA for sequencing. The inability to grow and sequence virus isolates is often a significant challenge for influenza surveillance in wild reservoirs and can be attributed to multiple factors.^46, 48, 49, 62^ As these animals appeared healthy at the time of sampling, it is possible that the viral load was too low for sustained viral shedding at a concentration required to propagate and sequence the virus, or that seal-derived IAV is poorly adapted to growth in ECEs. It is also possible that the strain(s) of virus circulating within the gray seal population are sufficiently divergent from avian isolates that the standardly used primers directed against untranslated regions of IAV are inadequate in this context. Alternatively, the inability to recover high concentrations of live virus could suggest that gray seals are a dead-end host that are frequently infected through avian cross-species transmission; as such they would not replicate nor shed high concentrations of virus.

As an alternative to molecular characterization, we utilized HI assays against sera from seropositive animals to subtype viruses. The HI assay provides a reasonable proxy of exposure based on antigenic cross-reactivity. Using this approach, we found that the gray seals in this study had a remarkably broad and diverse serologic profile against IAV, with some evidence of regional and temporal variations. The most frequently recognized subtypes were pH1N1, seal H3N8, H13 and H16. However, a large proportion of seropositive pups recognized H6, adults frequently recognized H2N3, and animals from Sable Island had a strong response to H4N6. Nearly all animals tested recognized the seal-derived H3N8 that was identified during the 2011 UME in harbor seals in the Gulf of Maine. This strongly suggests that although gray seals were not impacted by the UME, they were likely exposed and either H3N8 specific antibodies have been maintained for 4 years or more, or the virus continues to circulate. Also of particular interest, all groups of animals sampled on Cape Cod showed a moderate to strong reactivity against human pandemic H1N1; however animals from Sable Island did not. This is reminiscent of the recent report of the human population established pH1N1 infection found within northern elephant seals (*Mirounga angustirostris*) in California.^2^ Although this could be through direct interactions on coastal beaches, a more probable explanation postulated was that runoff from densely populated urban areas may contaminate coastal waters frequented by seals. The striking difference that we observed between a densely populated urban area (Eastern Massachusetts and Cape Cod) versus a remote island (Sable Island), lends further support to such an interpretation. Finally the observation that gray seals recognize H13 and H16 isolates is an intriguing one. The subtypes have almost exclusively been associated with *Anseriformes* (gulls and terns), with the one notable exception of a pilot whale (*Globicephala spp.)* H16N2 infection. The detection of these gull associated subtypes within gray seals suggests that the virus is competent for mammalian infection.^63^ It also highlights the potential connectivity between birds and coastal mammals in exchanging influenza.

Significant effort and resources are directed toward understanding IAV as it is an infectious agent that has caused, and continues to cause, substantial human morbidity and mortality. It is well established that IAV is maintained in wild bird populations and undergoes rapid and dramatic evolutionary changes through individual mutations and reassortment. Such changes occasionally result in viruses that are competent for mammalian transmission and can lead to threats of novel pandemic strains. The presence of a wild mammalian host that is repeatedly infected by IAV and potentially maintains virus at an endemic level could have a significant impact on how the virus evolves and adapts and is a critical component in understanding the overall disease ecology of IAV. The data reported here support the possibility that gray seals may serve as such a host, and warrants expanded analyses on these populations.

## Figures and Tables

**Table 1:** Overview of sampling effort from 2013 to 2015

**Season, year**	**Location**	**Sampled (virology)**	**Sampled (serology)**
Winter 2013	Cape Cod, USA	96 Pups	57 Pups
Summer 2013	Cape Cod, USA	15 Adults	12 Adults
Summer 2013	Sable Island, Canada	18 Adults	20 Adults
Winter 2014	Cape Cod, USA	103 Pups	99 Pups
Winter 2014	Sable Island, Canada	8 Adults	0
Winter 2014	Sable Island, Canada	20 Pups	0
Summer 2014	Sable Island, Canada	16 Adults	0
Winter 2015	Cape Cod, USA	126 Pups	113 Pups

## References

[bib1] Greig DJ. Health, disease, mortality and survival in wild, rehabilitated harbor seals (Phoca vitulina) inthe San Francisco Bay area and along the central California coast. PhD thesis, University of St Andrews, St Andrews, UK, 2011.

[bib2] Goldstein T, Mena I, Anthony SJ et al. Pandemic H1N1 influenza isolated from free-ranging Northern Elephant Seals in 2010 off the central California coast. PLoS One 2013; 8: 1–9. 10.1371/journal.pone.0062259 PMC3655164 23690933

[bib3] Anthony SJ, St Leger JA, Pugliares K et al. Emergence of fatal avian influenza in New England harbor seals. MBio 2012; 3: 1–9. 10.1128/mBio.00166-12 PMC3419516 22851656

[bib4] Blanc A, Ruchansky D, Clara M et al. Serologic evidence of influenza A and B viruses in South American fur seals (Arctocephalus australis). J Wildl Dis 2009; 45: 519–521. 19395764 10.7589/0090-3558-45.2.519

[bib5] Osterhaus ADME, Rimmelzwaan GF, Martina BEE, Bestebroer TM, Fouchier RAM. Influenza B virus in seals. Science 2000; 288: 1051–1053. 10807575 10.1126/science.288.5468.1051

[bib6] Ohishi K, Kishida N, Ninomiya A et al. Antibodies to human-related H3 influenza A virus in Baikal seals (Phoca sibirica) and ringed seals (Phoca hispida) in Russia. Microbiol Immunol 2004; 48: 905–909. 15557750 10.1111/j.1348-0421.2004.tb03610.x

[bib7] Webster RG, Hinshaw VS, Bean WJ et al. Characterization of an influenza A virus from seals. Virology 1981; 113: 712–724. 6267805 10.1016/0042-6822(81)90200-2

[bib8] Hinshaw VS, Bean WJ, Webster RG et al. Are seals frequently infected with avian influenza viruses ? J Virol 1984; 51: 863–865. 6471169 10.1128/jvi.51.3.863-865.1984 PMC255856

[bib9] Callan RJ, Early G, Kida H, Hinshaw VS. The appearance of H3 influenza viruses in seals. J Gen Virol 1995; 76: 199–203. 7844533 10.1099/0022-1317-76-1-199

[bib10] Runstadler J, Hill N, Hussein ITM, Puryear W, Keogh M. Connecting the study of wild influenza with the potential for pandemic disease. Infect Genet Evol 2013; 17: 162–187. 23541413 10.1016/j.meegid.2013.02.020 PMC3685205

[bib11] Zohari S, Neimanis A, Härkönen T, Moraeus C, Valarcher J. Avian influenza A(H10N7) virus involvement in mass mortality of harbour seals (Phoca vitulina) in Sweden, March through October 2014. Eurosurveillance 2014; 19: 1–6. 10.2807/1560-7917.es2014.19.46.20967 25425511

[bib12] Bodewes R, Bestebroer TM, van der Vries E et al. Avian influenza A(H10N7) virus-associated mass deaths among harbor seals. Emerg Infect Dis 2015; 21: 720–722. 25811303 10.3201/eid2104.141675 PMC4378483

[bib13] Krog JS, Hansen MS, Holm E et al. Influenza A(H10N7) virus in dead harbor seals, Denmark. Emerg Infect Dis J 2015; 21: 684–687. 10.3201/eid2104.141484 PMC4378493 25811098

[bib14] Fereidouni S, Munoz O, Von Dobschuetz S, De Nardi M. Influenza virus infection of marine mammals. Ecohealth 2016; 13: 161–170. 25231137 10.1007/s10393-014-0968-1

[bib15] Olsen B, Munster VJ, Wallensten A, Waldenström J, Osterhaus ADME et al. . Global patterns of influenza a virus in wild birds. Science 2006; 312: 384–388. 16627734 10.1126/science.1122438

[bib16] Bodewes R, García AR, Brasseur SM, Sanchez GJ. Seroprevalence of antibodies against seal influenza A (H10N7) virus in harbor seals and gray seals from the Netherlands. PLoS One 2015; 10: 1–9. 10.1371/journal.pone.0144899 PMC4684379 26658347

[bib17] Mandl JN, Ahmed R, Barreiro LB et al. Reservoir host immune responses to emerging zoonotic viruses. Cell 2014; 160: 20–35. 25533784 10.1016/j.cell.2014.12.003 PMC4390999

[bib18] Bean AGD, Baker ML, Stewart CR et al. Studying immunity to zoonotic diseases in the natural host - keeping it real. Nat Rev Immunol 2013; 13: 851–861. 24157573 10.1038/nri3551 PMC7098194

[bib19] Haydon DT, Cleaveland S, Taylor LH, Laurenson MK. Identifying reservoirs of infection : a conceptual and practical challenge. Emerg Infect Dis 2002; 8: 1468–1473. 12498665 10.3201/eid0812.010317 PMC2738515

[bib20] Peiris JSM, Poon LLM, Guan Y. Surveillance of animal influenza for pandemic preparedness. Science 2012; 335: 1173–1174. 22345402 10.1126/science.1219936

[bib21] Richard M, De Graaf M, Herfst S. Avian influenza A viruses: from zoonosis to pandemic. Future Virol 2014; 9: 513–524. 25214882 10.2217/fvl.14.30 PMC4157675

[bib22] Taubenberger JK, Morens DM. Influenza: the once and future pandemic. Public Health Rep 2010; 125: 16–26. PMC2862331 20568566

[bib23] Bowen WD, Den Heyer C, Hammil M. Pup production at Scotian Shelf Grey Seal (*Halichoerus grypus* colonies in 2010. Canadian Stock Assessment Secretariat. Can Sci Advis Secr Res Doc 2011; 2011/066: 1–31.

[bib24] Natural Environment Research CouncilScientific Advice on Matters Related to the Management of Seal Populations: 2014. SCOS: UK. 2014. Available at http://www.smru.st-andrews.ac.uk/documents/2589.pdf.

[bib25] Waring GT, Josephson E, Maze-Foley K, Rosel PE, editors. US Atlantic and Gulf of Mexico Marine Mammal Stock Assessments - 2014, 169–177. US: NOAA, 2015. Available at http://www.nefsc.noaa.gov/publications/tm/tm231/169_grayseal_F2014August.pdf.

[bib26] Roman J, Dunphy-Daly MM, Johnston DW, Read AJ. Lifting baselines to address the consequences of conservation success. Trends Ecol Evol 2015; 30: 299–302. 26042680 10.1016/j.tree.2015.04.003

[bib27] Johnston DW, Frungillo J, Smith A et al. Trends in stranding and by-catch rates of gray and harbor seals along the Northeastern coast of the United States: evidence of divergence in the abundance of two sympatric Phocid species? PLoS One 2015; 10: 1–12. 10.1371/journal.pone.0131660 PMC4511798 26200461

[bib28] Brasseur S, Czeck R, Diederichs B et al. Grey Seal surveys in the Wadden Sea and Helgoland in 2014-2015. Common Wadden Sea Secretariat: Wilhelmshaven, Germany, 2014. Available at http://www.waddensea-secretariat.org/sites/default/files/downloads/tmap/MarineMammals/GreySeals/grey_seal_report_2015.pdf.

[bib29] Rifkin JL, Nunn CL, Garamszegi LZ. Do animals living in larger groups experience greater parasitism? A meta-analysis. Am Nat 2012; 180: 70–82. 22673652 10.1086/666081

[bib30] Härkönen T, Dietz R, Reijnders P et al. The 1988 and 2002 phocine distemper virus epidemics in European harbour seals. Dis Aquat Organ 2006; 68: 115–130. 16532603 10.3354/dao068115

[bib31] Geraci JR, St Aubin DJ, Barker IK et al. Mass mortality of harbor seals: pneumonia associated with influenza A virus. Science 1982; 215: 1129–1131. 7063847 10.1126/science.7063847

[bib32] Boness DJ, James H. Reproductive behaviour of the Grey seal (*Halichoerus grypus* on Sable Island, Nova Scotia. J Zool 1979; 188: 477–500.

[bib33] Murray MJ. Behavioral Interactions Between Harbor Seals (Phoca vitulina) and Gray Seals (Halichoerus grypus) on Cape Cod, Massachusetts. Northeastern University: Boston, MA, USA. 2008. Available at http://hdl.handle.net/2047/d10016624.

[bib34] Thompson D, Onoufriou J, Bronlow A, Bishop A. Marine Mammal Scientific Support Research Programme MMSS/001/11. Preliminary report on predation by adult grey seals on grey seal pups as a possible explanation for corkscrew injury patterns seen in the unexplained seal deaths: addendum.. UK: Sea Mammal Research Unit. 2015. Available at http://www.smru.st-andrews.ac.uk/documents/scotgov/USD1and6_addendum_report_VF2.pdf.

[bib35] Van Neer A, Jensen LF, Siebert U. Grey seal (*Halichoerus grypus* predation on harbour seals (Phoca vitulina) on the island of Helgoland, Germany. J Sea Res 2015; 97: 1–4.

[bib36] Stringell T, Hill D, Rees D, Rees F, Rees P et al. . Short note: predation of harbour porpoises (*Phocoena phocoena* by grey seals (*Halichoerus grypus* in Wales. Aquat Mamm 2015; 41: 188–191.

[bib37] Haelters J, Kerckhof F, Jauniaux T, Degraer S. The grey seal (*Halichoerus grypus* as a predator of harbour porpoises (*Phocoena phocoena*? Aquat Mamm 2012; 38: 343–353.

[bib38] Grant DR, Bourne WRP. Grey Seals and Seabirds. The Seabird Group: Aberdeen. 1971-1972, pp. 52–53. Available at http://www.seabirdgroup.org.uk/journals/seabird_3.pdf.

[bib39] Lucas Z, McLaren IA. Apparent predation by grey seals, Halichoerus grypus, on seabirds around Sable Island, Nova Scotia. Can Field-Naturalist 1988; 102: 675–678.

[bib40] Bowen WD, McMillan J, Mohn R. Sustained exponential population growth of grey seals at Sable Island, Nova Scotia. ICES J Mar Sci 2003; 60: 1265–1274.

[bib41] Jessopp M, Cronin M, Hart T. Habitat-mediated dive behavior in free-ranging grey seals. PLoS One 2013; 8: 1–7. 10.1371/journal.pone.0063720 PMC3646810 23667663

[bib42] Lidgard DC, Bowen WD, Jonsen ID, Iverson SJ. Animal-borne acoustic transceivers reveal patterns of at-sea associations in an upper-trophic level predator. PLoS One 2012; 7: 1–8. 10.1371/journal.pone.0048962 PMC3498375 23155435

[bib43] Spackman E, Senne DA, Myers TJ et al. Development of a real-time reverse transcriptase PCR assay for type A influenza virus and the Avian H5 and H7 hemagglutinin subtypes development of a real-time reverse transcriptase PCR assay for type A influenza virus and the Avian H5 and H7 hemagglutini. J Clin Microbiol 2002; 40: 3256–3260. 12202562 10.1128/JCM.40.9.3256-3260.2002 PMC130722

[bib44] Slomka MJ, Densham ALE, Coward VJ et al. Real time reverse transcription (RRT)-polymerase chain reaction (PCR) methods for detection of pandemic (H1N1) 2009 influenza virus and European swine influenza A virus infections in pigs. Influenza Other Respi Viruses 2010; 4: 277–293. 10.1111/j.1750-2659.2010.00149.x PMC4634650 20716157

[bib45] USDA Wildlife ServicesUSDA/APHIS/Wildlife Services Instructions for NAHLN Laboratories Testing Wild Bird Samples. Manhattan: USDA. 2010. Available at https://www.aphis.usda.gov/wildlife_damage/nwdp/pdf/2010%20NAHLN%20labs%20testing%20WS%20wild%20bird%20samples.pdf.

[bib46] Munster VJ, Baas C, Lexmond P et al. Practical considerations for high-throughput influenza A virus surveillance studies of wild birds by use of molecular diagnostic tests. J Clin Microbiol 2009; 47: 666–673. 19109483 10.1128/JCM.01625-08 PMC2650931

[bib47] Moresco KA, Stallknecht DE, Swayne DE. Evaluation of different embryonating bird eggs and cell cultures for isolation efficiency of avian Influenza A Virus and Avian paramyxovirus serotype 1 from real-time reverse transcription polymerase chain reaction-positive wild bird surveillance samples. J Vet Diagn Invest 2012; 24: 563–567. 22529126 10.1177/1040638712440991

[bib48] Lindsay LL, Kelly TR, Plancarte M et al. Avian influenza: mixed infections and missing viruses. Viruses 2013; 5: 1964–1977. 23921843 10.3390/v5081964 PMC3761236

[bib49] Brown JD, Poulson R, Carter DL, Lebarbenchon C, Stallknecht DE. Infectivity of avian influenza virus-positive field samples for mallards: what do our diagnostic results mean? J Wildl Dis 2013; 49: 180–185. 23307386 10.7589/2011-11-322 PMC11373666

[bib50] Lin HT, Hsu CH, Tsai HJ et al. Influenza A plasma and serum virus antibody detection comparison in dogs using blocking enzyme-linked immunosorbent assay. Vet World 2015; 8: 580–583. 27047138 10.14202/vetworld.2015.580-583 PMC4774716

[bib51] Shriner SA, VanDalen KK, Root JJ, Sullivan HJ. Evaluation and optimization of a commercial blocking ELISA for detecting antibodies to influenza A virus for research and surveillance of mallards. J Virol Methods 2016; 228: 130–134. 26645952 10.1016/j.jviromet.2015.11.021

[bib52] Pederson JC. Hemagglutination inhibition test for avian influenza virus subtype identification and the detection and quantitation of serum antibodies to the avian influenza virus. In: Spackman E (ed). Avian Influenza Virus. Totowa, New Jersey: Humana Press; 2004, pp 53–66. 10.1007/978-1-59745-279-3_8 18370041

[bib53] Killian ML. Hemagglutination assay for the avian influenza virus. In: Spackman E (ed). Avian Influenza Virus. Totowa, New Jersey: Humana Press; 2004, pp 47–52.

[bib54] Cox N, Webster RG, Krauss S et al. WHO Manual on Animal Influenza Diagnosis and Surveillance, 2nd edn, Geneva: WHO. 2002. Available at http://www.who.int/csr/resources/publications/influenza/whocdscsrncs20025rev.pdf.

[bib55] Eriksson L, Andersson PL, Johansson E, Tysklind M. Megavariate analysis of environmental QSAR data. Part I - a basic framework founded on principal component analysis (PCA), partial least squares (PLS), and statistical molecular design (SMD). Mol Divers 2006; 10: 169–186. 16770514 10.1007/s11030-006-9024-6

[bib56] Saltelli A. Sensitivity analysis for importance assessment. Risk Anal 2002; 22: 579–590. 12088235 10.1111/0272-4332.00040

[bib57] Lehnert K, Müller S, Weirup L et al. Molecular biomarkers in grey seals (*Halichoerus grypus* to evaluate pollutant exposure, health and immune status. Mar Pollut Bull 2014; 88: 311–318. 25220314 10.1016/j.marpolbul.2014.08.025

[bib58] Beck CA, Bowen WD, McMillan JI, Iverson SJ. Sex differences in the diving behaviour of a size-dimorphic capital breeder: the grey seal. Anim Behav 2003; 66: 777–789.

[bib59] Lidgard DC, Boness DJ, Bowen WD, McMillan JI. Diving behaviour during the breeding season in the terrestrially breeding male grey seal: implications for alternative mating tactics. Can J Zool 2003; 81: 1025–1033.

[bib60] Webster RG, Bean WJ, Gorman OT, Chambers TM, Kawaoka Y. Evolution and ecology of influenza A viruses. Microbiol Rev 1992; 56: 152–179. 1579108 10.1128/mr.56.1.152-179.1992 PMC372859

[bib61] Wood SA, Frasier TR, McLeod BA et al. The genetics of recolonization: an analysis of the stock structure of grey seals (*Halichoerus grypus* in the northwest Atlantic. Can J Zool 2011; 89: 490–497.

[bib62] Stallknecht DE, Luttrell MP, Poulson R et al. Detection of avian influenza viruses from shorebirds: evaluation of surveillance and testing approaches. J Wildl Dis 2012; 48: 382–393. 22493113 10.7589/0090-3558-48.2.382 PMC3584701

[bib63] Hinshaw VS, Air GM, Gibbs AJ et al. Antigenic and genetic characterization of a novel hemagglutinin subtype of influenza A viruses from gulls. J Virol 1982; 42: 865–872. 7097861 10.1128/jvi.42.3.865-872.1982 PMC256920

